# Zone plates for angle-resolved photoelectron spectroscopy providing sub-micrometre resolution in the extreme ultraviolet regime

**DOI:** 10.1107/S1600577519000869

**Published:** 2019-02-15

**Authors:** Benedikt Rösner, Pavel Dudin, Jeroen Bosgra, Moritz Hoesch, Christian David

**Affiliations:** a Paul Scherrer Institut, 5232 Villigen PSI, Switzerland; b Diamond Light Source, Harwell Science and Innovation Campus, Didcot, Oxfordshire OX110DE, UK; c Photon Science, Deutsches Elektronen Synchrotron (DESY), Notkestrasse 85, Hamburg 22607, Germany

**Keywords:** angle-resolved photoelectron spectroscopy (ARPES), Fresnel zone plates, microspectroscopy, nanofocus

## Abstract

This article reports on the fabrication and testing of dedicated Fresnel zone plates for use at the nano-ARPES branch of the I05-ARPES beamline of Diamond Light Source to perform angle-resolved photoelectron spectroscopy with sub-micrometre resolution in real space. The aim of the design was to provide high photon flux combined with sub-micrometre spot sizes. The focusing lenses were tested with respect to efficiency and spatial resolution in the extreme ultraviolet between 50 eV and 90 eV. The experimentally determined diffraction efficiencies of the zone plates are as high as 8.6% at 80 eV, and a real-space resolution of 0.4 µm was demonstrated. Using the zone-plate-based setup, monolayer flakes of the two-dimensional semiconductor WS_2_ were investigated. This work demonstrates that the local electronic structure can be obtained from an area of a few micrometres across a two-dimensional heterostructure.

## Abstract 2

Fresnel zone plates for nano-ARPES in the extreme ultraviolet regime have been fabricated and tested. The optics provide high photon flux combined with spot sizes down to 0.4 µm.

## 1. Introduction   

Angle-resolved photoelectron spectroscopy (ARPES) has reached significant importance in modern condensed-matter physics (Damascelli, 2004 ▸; Lu *et al.*, 2012 ▸). Recording photoelectron spectra in an angle-resolved manner gives access to both binding energies below the Fermi edge and the momentum of probed electrons and thus the ability to map electronic states in three dimensions (Strocov *et al.*, 2014 ▸). With the capability to obtain information on the electron self-energy, the method provides ideal access to the electronic structure of strongly correlated systems. Typical phenomena which are successfully investigated with ARPES include superconductivity, magnetism, many-body correlation effects, or the influence of dopants or impurities on these properties (Lu *et al.*, 2012 ▸; Strocov *et al.*, 2014 ▸).

In view of the significance of this technique in scientific research, ARPES instruments can be found at various beamlines at almost every synchrotron radiation source in the world. In the most common geometry, X-rays are directed onto a sample surface, and the created photoelectrons are examined in hemispherical electron analysers that can be tilted to access the angular dependence of the photoemission. The X-ray spot size is typically in the range of 100 µm or larger, resulting in an average spectrum from a large area on the sample. In order to examine local defects, the band structure needs to be mapped not only in reciprocal space but also in real space. To this end, the incident photon beam needs to be focused down considerably (Kalläne *et al.*, 2011 ▸; Bostwick *et al.*, 2012 ▸). Concepts to obtain small focal spots for real-space ARPES mapping are possible, for example, at the nano-ARPES endstation of the I05 beamline at Diamond Light Source (Hoesch *et al.*, 2017 ▸), or similar dedicated endstations at Synchrotron Soleil (Avila *et al.*, 2013 ▸) and the Advanced Light Source (Bostwick *et al.*, 2012 ▸). The scientific case includes two-dimensional materials, topological insulators, superconductors, strongly correlated materials and *operando* studies of micro-devices (Khalil *et al.*, 2017 ▸).

Compared with other means of focusing X-rays, Fresnel zone-plate (FZP) lenses offer some particular advantages for application in ARPES: they are easy to align and they produce a very defined, reliable focal spot. At the I05 beamline, we aim to focus the extreme ultraviolet (EUV) photon beam into a spot with a size well below 1 µm, ideally down to a few hundred nanometres. Assuming the diffraction limit from the Abbe equation (Abbe, 1873 ▸) and the simplified expression for the numerical aperture (NA) according to the zone-plate equation (1), we can derive the diffraction-limited spot size *s* from the width of the outermost zone *dr*,
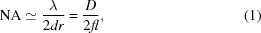


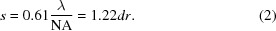
A couple of restrictions apply when employing FZP lenses for ARPES in the EUV regime. Firstly, the vicinity of the sample has to be free of material which disturbs the trajectory of electrons in order to exclude any influence on the photoelectrons under investigation. Secondly, the sample surface is oriented with an angle of 45° to the incident photon beam. Both restrictions result in the requirement for large gaps between the sample and the order-sorting aperture (OSA), which is required to block the higher diffraction orders of the FZP. We consider a separation between the OSA and sample of 4 mm as the minimum for the experimental geometry at I05. This distance is achieved using FZPs with a minimum focal length of 8 mm, a zone-plate diameter of 1.5 mm and an OSA diameter of half of that value. In addition, a central beam stop has to be used to block the undiffracted light which transmits through the lens, with a diameter that is slightly larger than the OSA, thus allowing for alignment mismatches.

With a zone-plate diameter of 1.5 mm and a photon energy of 50 eV (λ = 24.8 nm), a minimum focal length of 8 mm induces a limit of 132 nm for the outermost zone width. The use of a FZP with smaller zones either decreases the focal length with the risk of influencing electrons by correspondingly reducing the distance between the OSA and sample, or requires FZPs with larger diameters and more zones. Larger diameters lead to less demagnification, imposing more stringent preconditions in terms of spatial coherence, and a higher number of zones further lowers the required energy bandwidth of the incident photon beam. The parameters presented above present a good compromise between a preferably small spot size and the optics geometry of the I05 beamline for an energy range between 50 eV and 90 eV.

Another challenge is the high absorption in the EUV regime of commonly used support membrane materials. The material choice thus plays a major role in FZP fabrication. As silicon nitride has a high EUV absorption, it has to be either ultrathin, *i.e.* ≤50 nm, or silicon membranes have to used instead of silicon nitride.

In this study, we present the fabrication of FZP lenses dedicated to spatially resolved ARPES at the I05 beamline in the EUV regime. We present details about the materials used, processing parameters and resulting nanostructures. The fabricated zone plates have been thoroughly tested and their performance in terms of spot size and efficiency has been characterized. The challenges of using our lenses in nano-ARPES applications are discussed in light of the experimental results.

## 2. Zone plate fabrication   

Fresnel zone plates for nano-ARPES were fabricated on substrate materials that exhibit reasonably low absorption in the EUV regime. For the first set of zone plates, we fabricated silicon nitride membranes with a 1.7 mm × 1.7 mm lateral dimension from wafers coated with 28 nm-thick silicon nitride by a potassium hydroxide wet-etch process. Special attention must be paid during processing and handling to avoid breakage of these ultrathin membranes. In more recent fabrication, newly available silicon membranes from Norcada Inc. were used (CUF521.7D). These membranes are 200 nm thick, circular with a diameter of 1.7 mm and are flattened by a special frame. This frame induces tensile stress to prevent wrinkling which usually occurs on membranes of bare silicon (for more information, see http://www.norcada.com/products/silicon-membranes/). The EUV transmission of the 200 nm-thick silicon substrate is comparable with the 28 nm-thick silicon nitride membranes (Henke *et al.*, 1993 ▸); however, they are much more robust.

Hydrogen silsesquioxane (HSQ, Fox 16, diluted 1:2 with methyl iso­butyl ketone) was spun onto the membranes at 2000–4000 rpm, resulting in a coating of 150–250 nm thickness. The HSQ was then exposed in a Vistec EBPG 5000+ electron lithography instrument operated at 100 kV. Typical beam currents ranged between 5 nA and 20 nA. Subsequently, the exposed resist was developed in a 1:3 mixture of AZ 351 developer and water, then rinsed with water and iso­propanol.

The fabricated zone plates have a diameter of 1.5 mm and an outermost zone width of 132 nm. The resulting focal length is between 8 mm and 14.4 mm for the energy range of 50 eV to 90 eV. The prepared zone plates were then mounted on tungsten chips with laser-cut central stops of 750 µm diameter under an optical microscope. The beam stop size ensures that the smallest gap between the OSA (diameter of 700 µm) and sample is 4 mm at 50 eV to avoid parasitic influence from the OSA to the kinetic energy and momentum of the photoelectrons.

**Figure 1:**
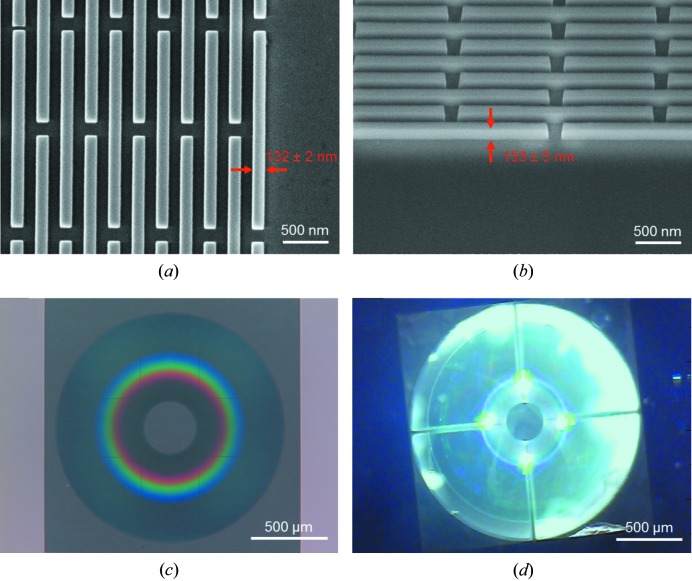
(*a*) Scanning electron micrograph of the outermost zones of the Fresnel zone plate taken under normal incidence. The outermost zone width corresponds to the desired 132 nm. (*b*) The outermost zones viewed at a 45° tilt angle. The height of the zone plate is 153 ± 5 nm, yielding the maximum possible efficiency at 70 eV. (*c*) Optical micrograph of the Fresnel zone plate, showing that the structures are clean and regular. The vertical and horizontal lines correspond to the stitching of main fields in the electron beam lithography tool. (*d*) Fresnel zone plate mounted onto the chip with a laser-cut central beam stop.

Figs. 1 ▸(*a*) and 1 ▸(*b*) show the resulting structures recorded using a scanning electron microscope (SEM). The micrographs confirm a high structural quality of the zone plate with an exact 1:1 ratio for the widths of silicon oxide zones and gaps [132 ± 2 nm, see Fig. 1 ▸(*a*)]. The height is 153 ± 5 nm, which gives an ideal diffraction efficiency for use at 70 eV [Fig. 1 ▸(*b*)]. The whole zone plate appears clean and regular, with the borders of the main fields of the lithography system visible as sharp lines under the optical microscope [Fig. 1 ▸(*c*)]. A closer look at these regions in the SEM confirms that the high structural quality is not negatively affected by writing field stitching in the lithography step, with displacements small enough that they do not affect the zone-plate performance. Fig. 1 ▸(*d*) shows the zone plate after mounting on the chip with the central beam stop.

## 3. Performance of the Fresnel zone plates   

In ARPES, the data quality is significantly limited by the number of photons available on the sample. The theoretical efficiency of the setup using the zone plate compared with the beamline flux without zone plate optics is calculated to be between 4 × 10^−3^ and 9 × 10^−3^. The given efficiency comprises at least three factors: the light which is lost due to the zone-plate aperture and central stop, the absorption of the support membrane and the diffraction efficiency of the zone plate itself. The zone plate is illuminated with a Gaussian-shaped beam, cutting off parts of the radiation outside the aperture. At a photon energy of 50 eV, the Gaussian beam shape has a full width at half-maximum (FWHM) of approximately 2 mm in the horizontal and 6 mm in the vertical direction according to the results of ray tracing. These figures correspond well to the sizes of light spots observed on the screen placed about 10 cm after the zone plate. The remaining fraction can be simulated with ray-tracing software like *SHADOW* or *RAY*, and lies in the range 5–10% depending on the photon energy. The diffraction efficiency of the zone plate including the absorption of its substrate at 80 eV has been calculated to be 8.6% in the first order with rigorous coupled wave theory (Schneider, 1997 ▸). Although a precise efficiency measurement is difficult with this setup, the total efficiency of the setup was determined to be 0.16%. This figure is determined experimentally using an alternative focusing mirror (with a larger spot size), which focuses the complete incoming flux onto the sample. The resulting flux at the sample is up to 1.5 × 10^10^ photons s^−1^ when the synchrotron is operated with a 300 mA ring current. This is sufficient for ARPES experiments with an energy resolution of 30–50 meV.

**Figure 2:**
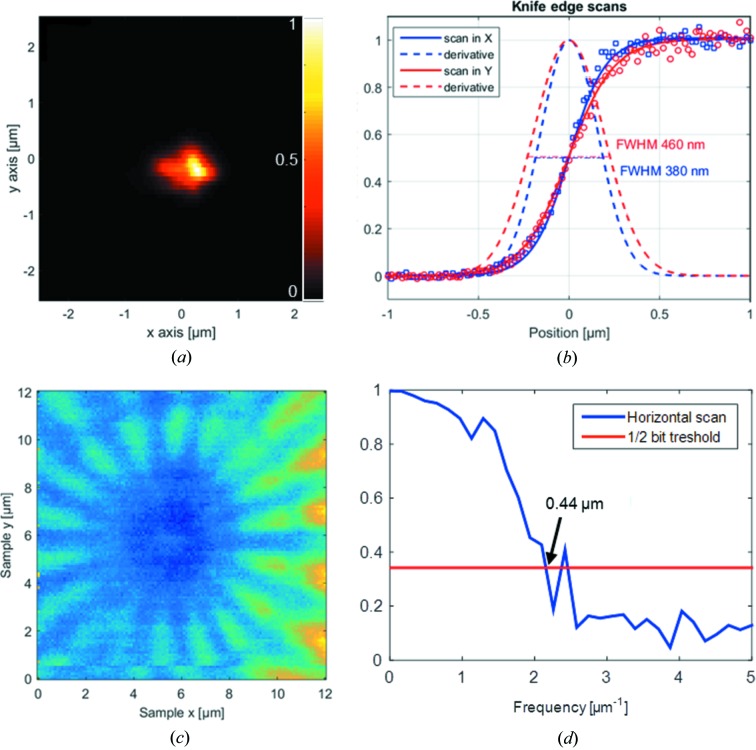
(*a*) Spot profile in focus at a photon energy of 80 eV as reconstructed from an exposed and solvent-treated PMMA film. The beam intensity is linear with the colour scale. (*b*) Knife-edge scan in the horizontal (blue) and vertical (red) direction. Dots represent the experimental data; the solid lines are fit with the error function, supposing a Gaussian shape of the first derivative (dashed lines). Its FWHM is 0.38 µm and 0.46 µm. (*c*) Scanning micrograph of a Siemens Star test pattern recorded at 70 eV in 100 nm-wide steps. (*d*) One-dimensional Fourier shell correlation of the image depicted in (*c*). The frequency cut-off in the horizontal scanning direction corresponds to 0.44 µm.

In order to obtain a detailed picture of the intensity distribution in the focal plane, we exposed a 1 µm-thick film of poly(methyl)­methacrylate (PMMA) to the beam in focus of the FZP. A similar technique based on polymer imprints is frequently used to characterize the beam profile at free-electron lasers (Chalupský *et al.*, 2010 ▸) and has been refined by treating the exposed polymer with organic solvents similar to methods in lithography (Rösner *et al.*, 2017 ▸). Although synchrotron radiation is not capable of creating imprints directly (due to the lower number of photons per second), the photon dose required to remove the polymer can be accumulated on a longer timescale. From the Beer–Lambert law (Beer, 1852 ▸) and the attenuation length of the EUV radiation in PMMA, we are able to reconstruct a map of the beam intensity from the local depth of the craters which were created by solvent-treatment of the polymers. Fig. 2 ▸(*a*) shows the reconstructed beam intensity in the focal plane. For this measurement, the photon energy was set to 80 eV, and a series of spots was exposed at different focal distances. The polymer film was then treated with organic solvents in a procedure described in detail elsewhere (Rösner *et al.*, 2017 ▸). The resulting craters were analysed with using atomic force microscopy (AFM).

The cross-section of the imprint shows similar dimensions as knife-edge scans, which were performed at the edge of a membrane using a diode behind the sample plane to detect the transmitted intensity [Fig. 2 ▸(*b*)]. Note that the side peak in the *x* direction is present at all imprints and can be explained as a result of sample positioning which was carried out in the *x* direction with an open beam shutter. The FWHM of the first derivative of the knife-edge scans in the focal plane is 0.38 µm in the horizontal and 0.46 µm in the vertical scanning direction.

To confirm the spatial resolution for ARPES applications, we scanned a Siemens star test pattern. The Siemens star consists of 30 nm-thick silicon oxide deposited on a 15 nm-thick iridium film. The innermost width of its spokes is 80 nm (periodicity of 160 nm). An area of 12 µm × 12 µm was scanned with a step size of 100 nm. Fig. 2 ▸(*c*) shows the resulting intensity map of photoelectrons. Clearly visible structures, 1 µm in size, are nicely resolved. Note that the Siemens star pattern includes an additional ring to mark the feature size of 1 µm. Inside this ring, lines and spaces are still visible, albeit with reduced contrast. To evaluate the limitation of the spatial resolution quantitatively, we conducted a one-dimensional Fourier shell correlation (van Heel & Schatz, 2005 ▸; Mohacsi *et al.*, 2017 ▸). The frequency cut-off using the half-bit criterion is determined to be 2.3 µm^−1^, corresponding to a 0.44 µm resolution in real space. An analysis in the vertical direction yields similar values. These values are in good agreement with the findings from the knife-edge scans and the beam profiling with PMMA, indicating that ARPES scans can be conducted with spatial resolutions below 0.5 µm.

**Figure 3:**
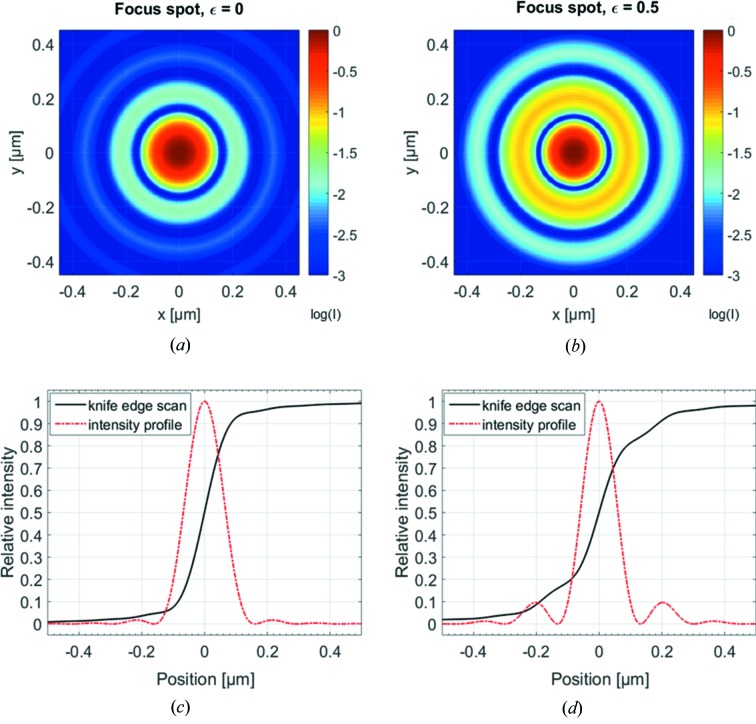
Simulated diffraction-limited spot of a Fresnel zone plate with an outermost zone width of 132 nm. (*a*) Without (∊ = 0) and (*b*) with a central beam stop with half the diameter of the zone plate (∊ = 0.5). The intensity is normalized to the maximum intensity in the centre and visualized on a logarithmic scale. (*c*) Calculated intensity of a knife-edge scan through (*a*) in black and its intensity profile (red dashed line), and (*d*) the corresponding knife-edge profile for (*b*) in black and its intensity profile (red dashed line).

To obtain an estimate on the theoretical spot size and shape, we calculated the diffraction-limited spot of our FZPs. The intensity distribution in the focal plane can be described with a first-order Bessel function, taking into account the outermost zone width *dr* and the ratio *∊* of the diameters of the central beam stop and zone plate (Born & Wolf, 1970 ▸),
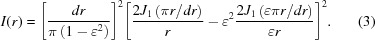
Although the majority of the total intensity is found at the optical axis when no beam stop is present, as shown in Fig. 3 ▸(*a*), the intensity distribution looks different for 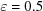, *i.e.* with a central stop with half the diameter of the zone plate [Fig. 3 ▸(*b*)]. Looking at the radial intensity integral, *i.e.* the intensity fraction within a certain distance from the optical axis, a striking difference becomes obvious. While 86% of the total intensity is found within a radius that corresponds to the Rayleigh criterion of 1.22*dr* = 160 nm lacking a central stop, merely 51% of the total intensity falls within this radius when a beam stop with half of the diameter of the FZP is used. The radius containing 85% of the radiation exceeds 250 nm in this case, explaining the comparably large spot size found in resolution tests. The difference becomes even more evident when a knife-edge scan is calculated [see Figs. 3 ▸(*c*) and 3(*d*)]. Although the curve has a quite steep slope when no central stop is present, it smears out over a large range with central stop. The simulated knife-edge scan with the central stop is significantly closer to the real knife-edge profile as shown in Fig. 2 ▸(*b*).

**Figure 4:**
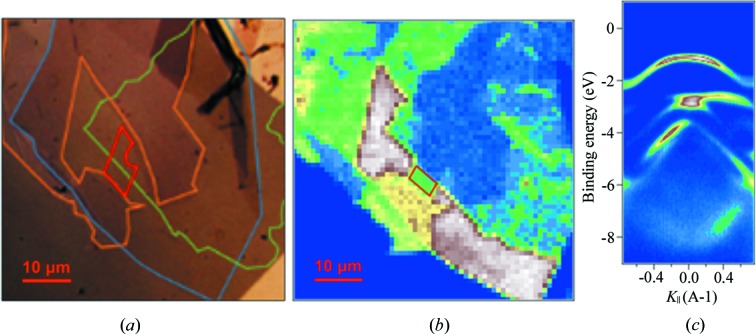
Spatially resolved ARPES of graphene grown on a heterostructure of two-dimensional materials, courtesy of N. Wilson and N. Teutsch at the University of Warwick. (*a*) Optical micrograph of the heterostructure with outlined borders of the layers. The bottom layer is BN (blue) covered with multilayer WS_2_ (orange) and monolayer WS_2_ (red) at the same level. A graphene layer (green) is on top of the heterostructure. (*b*) Total electron yield map from the integrated intensity on the two-dimensional detector. The red rectangle outlines the few micrometres area where the flake consisting of monolayer WS_2_ is isolated without any other layer on top or below. The contrast of the image is the result of interplay between electronic dispersions of the materials composing the heterostructure. (*c*) High-symmetry cut from electronic dispersion measured on the area indicated in (*b*) at a photon energy of 70 eV.

To test the performance of our setup for ARPES applications, we used a heterostructure of two-dimensional materials. The high electron mobility observed in two-dimensional materials makes it a prospective material for field-effect transistors (Wilson & Yoffe, 1969 ▸). The heterostructure we studied consists of graphene, a monolayer of tungsten di­sulfide (WS_2_) and a monolayer of boron nitride (BN) as a model system. The sheets were exfoliated in a simple approach using adhesive tape, resulting in multiple flakes with suitable dimensions for nano-ARPES applications. Fig. 4 ▸ shows a prototype heterostructure for a field-effect transistor as imaged in the optical microscope [Fig. 4(*a*)] and in total electron yield [Fig. 4(*b*)]. An area was carefully chosen representing a monolayer flake of WS_2_ that is only a few micrometres in size [red square in Fig. 4 ▸(*b*)]. At each point on the sample surface, we can measure the three-dimensional distribution of photo-emitted electrons by direction and kinetic energy. Fig. 4 ▸(*c*) shows a slice of the three-dimensional ARPES spectrum of the chosen region in one direction of the *k*-space. In this way, the local dispersion of electrons can be selectively probed from a defined area of a few micrometres.

## 4. Conclusions   

The FZPs described here were designed for use at the nano-ARPES branch of the I05-ARPES beamline of Diamond Light Source, with emphasis on high photon flux in the EUV regime and the possibility to achieve a spot size as small as possible. The design and material choice are mainly driven by the high flux required of the ARPES application, making nanofabrication and handling of the lenses extremely demanding. Although the design has been optimized to meet this purpose, the photon-hungry spectroscopy method is commonly performed under conditions where a diffraction-limited spot size is not achieved. Furthermore, the diffraction-limited spot is effectively broadened by the use of the central stop, and the spot is thus more than twice as large as the Rayleigh criterion suggests. These FZPs enable experiments of spatially resolved ARPES routinely at spot sizes well below 1 µm even under more relaxed conditions of everyday data acquisition, *i.e.* a larger exit-slit opening for an improved signal-to-noise ratio.

Spatial resolution below 1 µm is a remarkable achievement in ARPES; this will enable investigations of a multitude of material systems where the electronic structures vary on the surface. This comprises, for example, though not exclusively, patterned semiconductors, materials with interfaces, patterned samples or electronic effects at defects. Moreover, the large working distance favours convenient work with an electron energy analyser by minimizing the possible distortion of the electric field by OSA.
